# Curcumin induces crosstalk between autophagy and apoptosis mediated by calcium release from the endoplasmic reticulum, lysosomal destabilization and mitochondrial events

**DOI:** 10.1038/cddiscovery.2015.17

**Published:** 2015-10-26

**Authors:** A Moustapha, PA Pérétout, NE Rainey, F Sureau, M Geze, J-M Petit, E Dewailly, C Slomianny, PX Petit

**Affiliations:** 1 INSERM U1124 ‘Toxicologie, Pharmacologie et Signalisation Cellulaire’, Université Paris-Descartes, Centre Universitaire des Saints-Pères, Paris, France; 2 Université Pierre et Marie Curie—Paris 6, Laboratoire Jean Perrin, Paris, France; 3 Muséum National d’Histoire Naturelles, CeMIM/USM 0504, ‘Biologie Fonctionnelles des Protozoaires’ 57, Paris, France; 4 Muséum National d’Histoire Naturelles, UMR 7245 CNRS/MNHN ‘Molécules de Communication et Adaptation des Micro-organismes’ 57, Paris, France; 5 Laboratoire de Physiologie cellulaire, INSERM U800, Université des Sciences et Techniques de Lille 1, Villeneuve d'Ascq, France

## Abstract

Curcumin, a major active component of turmeric (*Curcuma longa*, L.), has anticancer effects. *In vitro* studies suggest that curcumin inhibits cancer cell growth by activating apoptosis, but the mechanism underlying these effects is still unclear. Here, we investigated the mechanisms leading to apoptosis in curcumin-treated cells. Curcumin induced endoplasmic reticulum stress causing calcium release, with a destabilization of the mitochondrial compartment resulting in apoptosis. These events were also associated with lysosomal membrane permeabilization and of caspase-8 activation, mediated by cathepsins and calpains, leading to Bid cleavage. Truncated tBid disrupts mitochondrial homeostasis and enhance apoptosis. We followed the induction of autophagy, marked by the formation of autophagosomes, by staining with acridine orange in cells exposed curcumin. At this concentration, only the early events of apoptosis (initial mitochondrial destabilization with any other manifestations) were detectable. Western blotting demonstrated the conversion of LC3-I to LC3-II (light chain 3), a marker of active autophagosome formation. We also found that the production of reactive oxygen species and formation of autophagosomes following curcumin treatment was almost completely blocked by *N*-acetylcystein, the mitochondrial specific antioxidants MitoQ10 and SKQ1, the calcium chelators, EGTA-AM or BAPTA-AM, and the mitochondrial calcium uniporter inhibitor, ruthenium red. Curcumin-induced autophagy failed to rescue all cells and most cells underwent type II cell death following the initial autophagic processes. All together, these data imply a fail-secure mechanism regulated by autophagy in the action of curcumin, suggesting a therapeutic potential for curcumin. Offering a novel and effective strategy for the treatment of malignant cells.

## Introduction

Curcumin, extracted from the plant *Curcuma Longa*, has long been recognized as a very promising anticancer drug because of its many beneficial properties, including anti-inflammatory, anti-oxidant and anti-carcinogenic activities.^1,2^ Curcumin is a potent bioactive compound that is used to treat cancer,^3^ atherosclerosis^4^ and neurodegenerative diseases, such as Alzheimer’s^4,5^ and Parkinson’s disease.^6^ Curcumin is particularly appealing as a therapeutic agent because of its low toxicity, although its intracellular bioavailability is quite low.^7^ Curcumin has anti-proliferative and anti-carcinogenic properties and may be useful for cancer prevention. The mechanisms underlying curcumin-induced cancer cell death have not been clearly defined, although available evidence suggests that curcumin downregulates NF-*κ*B signaling, which suppresses proliferation and induces apoptosis.^8,9^ Although both caspase 8-mediated and/or caspase 9-dependent apoptosis have been reported to occur upon the exposure of cancer cells to curcumin,^10,11^ discrepant results exist for caspase-8.^12^ Indeed, it is still unclear which caspase functions as the initiator caspase. Curcumin induces mitochondria-mediated apoptosis^13,14^ and reactive oxygen species (ROS) have been implicated in this process.^15^ Complex interplay between autophagy and apoptosis has come to light. Autophagy is involved in the anti-proliferative and apoptotic activities of curcumin,^16^ both *in vitro* and *in vivo*. Curcumin inhibit the growth of malignant cells by activating autophagic-cell death via the Akt/mTOR/p70S6K signaling and ERK1/2 pathways.^17^

Here, we show that curcumin with its multifaceted action induces different cell death pathways together with autophagy in a dose-dependent relationship. An intricated crosstalk between autophagy and apoptosis is initiated that correspond to the hormesis characteristics of curcumin. We discuss these results in light of the recent use of curcumin as an anticancer therapy.

## Results

### Cellular uptake and intracellular distribution of curcumin

**Figure 1:**
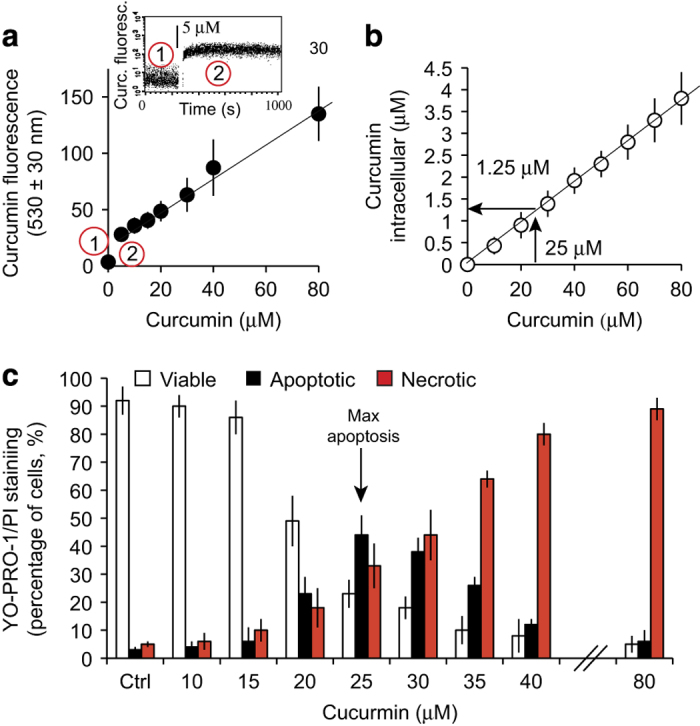
Cellular uptake and intracellular concentration of curcumin and induction of cell death. (**a**) Flow cytometry measurement of curcumin fluorescence in Huh-7 cells treated with different amounts of curcumin (0–80 *μ*M external concentration). In the upper panel 1 shows the autofluorescence of cells at 530±30 nM (flavoprotein fluorescence), and 2 shows the fluorescence after the addition of a defined amount of curcumin (5 *μ*M). The plotted mean values were recorded after 5 min of equilibration. (**b**). Intracellular curcumin concentration in *μ*M, as measured by methanolic extraction, of cells treated with various concentrations of curcumin plotted against the external concentration of curcumin (0–80 *μ*M; *n*=6 independent experiments). Huh-7 cells were used as negative controls (to determine cellular autofluorescence at the wavelength used, i.e., 530±30 nm). (**c**) Percentage of viable, apoptotic and necrotic cells among cells treated with various concentrations of curcumin for 24 h. YO-PRO-1/PI staining was used to analyze the three populations: YO-PRO-1^−^/PI^−^ for viable cells, YO-PRO-1^+^/PI^−^ or YO-PRO-1^+^/PI^intermediate^ for apoptotic cells and YO-PRO-1^+^/PI^+^ for necrotic cells. The maximum of proportion of apoptotic cells is observed at 25 *μ*M of curcumin for 24 h. Data are expressed as the mean±S.D. (*n*=10).

We incubated Huh-7 cells with curcumin (up to 80 *μ*M) for 5 min and measured its uptake (cellular viability: 96%). There was a linear relationship between curcumin concentration and cellular fluorescence (Figure 1a).

**Figure 2:**
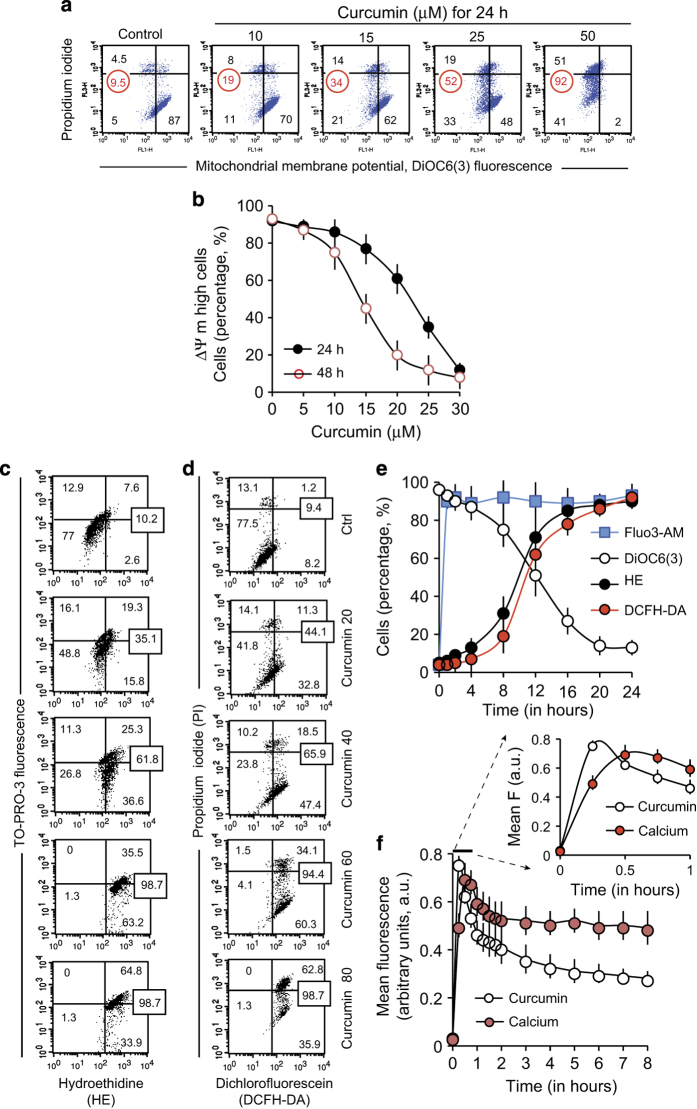
Flow cytometry analysis of the events linked to curcumin-induced cell death. (**a** and **b**) Flow cytometry analysis of the mitochondrial membrane potential (ΔΨ_m_) and membrane integrity by double staining with 20 nM 3,3’-dihexyloxacarbocyanine iodide (from a 10 *μ*M stock solution in ethanol) and 1 *μ*g/ml PI (from a 1 mg/ml stock solution). (**c** and **d**) Percentage of cells producing ROS after treatment with various concentrations of curcumin (0–80 *μ*M). Superoxide anion production (**c**) as measured by the hydroethidine staining, and hydrogen peroxide production (**d**) as measured by the dichlorofluorescein fluorescence of the DCFH-DA (dichlorofluorescein diacetate). All measurements were made in presence of PI to discriminate between live and dead cells. (**e**). A 24 h time course of ΔΨ_m_, calcium content (with Fluo3-AM) and superoxide anion and hydroperoxide production in cells treated with 25 *μ*M of curcumin. (**f**). A 8-h time course showing the rapid uptake of curcumin followed by an immediate increase in calcium levels. In all figures, when a single experiment is shown as an example, the data are expressed as a percentage of the whole population, whereas in concentration or time scale curves, the data are either expressed as mean fluorescence (in arbitrary units)±S.D. or as a percentage (%)±S.D. with *n*=9.

We next measured intracellular curcumin concentration that was much lower than the extracellular curcumin, although the two measures were linearly related (Figure 1b). For external curcumin at 25 *μ*M, the intracellular curcumin is of 1.25 *μ*M (ratio 20/1). Curcumin penetrated the cells rapidly: within 5 min a significant amount of curcumin was found within the cells (at 5 *μ*M; Figure 1a upper left panel). Intracellular curcumin levels were maximal within 10 min and decreased slowly over the following hours (Figure 2f).

### Curcumin induces apoptosis and necrosis and an increase in mitochondrial membrane potential, ROS production and calcium levels

After incubation with curcumin, cells were double stained with YO-PRO-1 (Y)/propidium iodide (PI) and analyzed by flow cytometry to discriminate between viable (Y^−^/PI^−^), apoptotic (Y^+^/PI^−^) and necrotic (Y^+^/PI^−+^) cells (Figure 1c). Curcumin concentrations of 20–25 *μ*M induced apoptosis, whereas higher concentrations favored necrosis over apoptosis. And as the curcumin concentration increases, apoptosis is progressively replaced with necrosis, probably because curcumin is cytotoxic at high concentrations (≥25 *μ*M).

Analysis of the mitochondrial membrane potential (ΔΨ_m_) and viability (PI; Figure 2a) revealed that curcumin decreased the ΔΨ_m_ after 24 h of incubation in viable cells. Indeed, at 15 *μ*M of curcumin, 21% of viable PI^−^ cells showed a low ΔΨ_m_ as opposed to only 5% of viable cells with low potential in untreated samples. This was associated with a small proportion of dead (PI^+^) cells, which was 4.5% in untreated conditions and 14% at 15 *μ*M of curcumin. At curcumin concentrations of 25 *μ*M, more than half of cells had a low ΔΨ_m_ (52%). Moreover, cells started to become permeabilized (high intermediate PI^−/+^ staining and PI^+^ staining) and the proportion of PI^+++^ cells with a low ΔΨ_m_ was 19%. At 50 *μ*M of curcumin, 51% of PI^+^ cells and 92% of all cells had a low ΔΨ_m_. Longer incubation time (48 h) resulted in fewer viable cells exhibiting a high ΔΨ_m_ (Figure 2b).

In general, a ΔΨ_m_ drop is associated with ROS production resulting from mitochondrial destabilization.^18^ We therefore assayed superoxide anion (O^−^) and hydroperoxide (H_2_O_2_) production in curcumin-treated cells (Figures 2c and d). Consistent with a mitochondrially induced apoptotic pathway, the decline in ΔΨ_m_ was associated with a raise of O^−^ and H_2_O_2_ (Figures 2a and b). ROS production increased as a function of incubation time (Figure 2e). Experiments involving diverse anti-oxidants, including *N*-acetylcysteine (NAC), Trolox, vitamin E and the mitochondrially targeted MitoQ10 or SQK1, indicated that ROS were produced by mitochondria (Supplementary Figure S2). MitoQ10 or SKQ1, which specifically target the mitochondrial matrix, were more effective than NAC and Trolox at limiting ROS levels.

These data and calcium fluorescence measured with Fluo3-AM are plotted as a timecourse for cells treated with 25 *μ*M curcumin for up to 24 h (Figures 2e and f). After curcumin treatment, there was an increase in the calcium concentration followed by a ΔΨ_m_ loss and ROS production (Figure 2f). Thus, curcumin enters Huh-7 cells rapidly (Figure 2f) where it provokes a calcium pulse and the early apoptotic events, followed by a drop in ΔΨ_m_ and an increase in ROS production (Figure 2e). Cellular calcium content appeared to be linked to curcumin fluorescence (Figure 2f). Indeed, both decreased similarly as a function of time after an initial peak in concentration within the first minutes of curcumin addition. Closer inspection of the early time points reveals that curcumin fluorescence increased before calcium fluorescence increased (Figure 2f enlargement). We therefore investigated the mechanisms of the calcium increase that rapidly follows curcumin uptake and precedes the mitochondrial events (Figure 2e).

### Opening of the mitochondrial transition pore in isolated mitochondria

**Figure 3:**
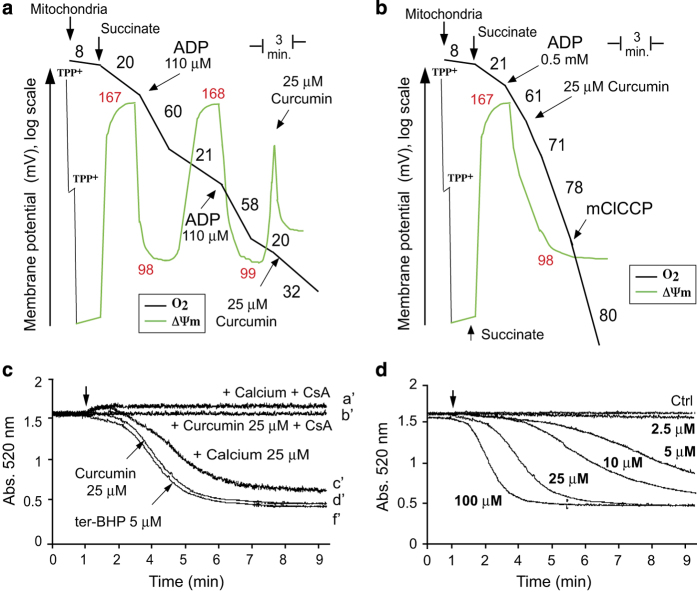
Effect of curcumin on mitochondrial bioenergetics and mitochondrial PTP opening. (**a**) Trace control. Recordings of ΔΨ_m_ (TPP^+^ electrode measurements; green line) and oxygen consumption (Clark electrode; black line) in purified mouse liver mitochondria. The numbers in black along the trace show nmol O_2_/min/mg protein and the ΔΨ_m_ is reported in mV. TPP^+^ was added to calibrate the electrode. Measurements were performed in the presence of succinate (1 mM) and rotenone (5 *μ*M) together with a saturating amount of ADP (0.5 mM). The uncoupler, mClCCP (10 *μ*M), was added at the end of the trace to uncouple fully respiration. (**b**) Effect of 25 *μ*M curcumin on ΔΨ_m_ (TPP^+^ electrode measurements; green line) and oxygen consumption (Clark electrode; black line) in purified mouse liver mitochondria. The experiment was performed in the same conditions as for trace **a**. (**c**) Opening of the mitochondrial permeability transition pore in different conditions; comparison between PTP opening with 25 *μ*M calcium, 5 *μ*M ter-butylhydroperoxide or 25 *μ*M curcumin. CsA was used at 5 *μ*M. One representative experiment (*n*=6) is shown. (**d**) Opening of the mitochondrial permeability transition pore in response to increasing concentrations of curcumin (from 2.5 to 100 *μ*M).

As it has been demonstrated that isolated mitochondria where sensitive to curcumin we tried to test this in our conditions. We incubated a mitochondrial suspension with 25 *μ*M of curcumin. This inhibited state 3 respiration (Figure 3a), uncoupled state 4 respiration and led to a drop in ΔΨ_m_ in state 4 (Figure 3b). We tested permeability transition pore (PTP) opening in presence of 25 *μ*M curcumin (Figure 3c). Calcium induces the opening of the PTP in a similar fashion as ter-butylhydroperoxide and pore opening is inhibited by cyclosporin A (Figures 3c and d). The presence of 25 *μ*M curcumin opened the PTP, but the initial mitochondrial shrinkage associated with calcium uptake was not observed and the curve of PTP opening was identical to that in the presence of 5 *μ*M of ter-butylhydroperoxide. The minimal curcumin concentration that caused the PTP to open in isolated mitochondria was ≥2.5 *μ*M (Figure 3d); higher concentrations promoted PTP opening and slightly modified the kinetics of opening (Figure 3d).

### Curcumin treatment leads to mitochondrial swelling, cytochrome *c* release and the accumulation of autophagic vacuoles *in vitro*

**Figure 4:**
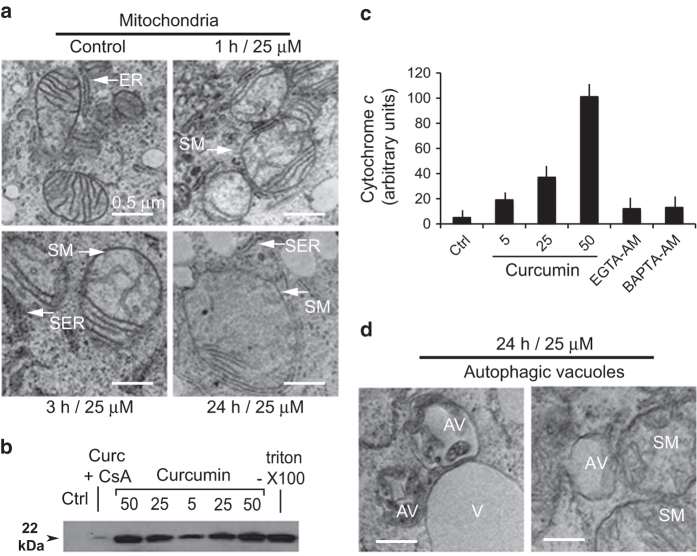
Electron microscopy of mitochondria and cytoplasm of curcumin-treated Huh-7 cells. (**a**) Electron micrographs illustrating time-dependent mitochondrial swelling in cells incubated with 25 *μ*M curcumin for 1, 3 or 24 h. (**b** and **c**) Western blots of cytochrome *c* released in cells treated with different concentrations of curcumin, in the presence or absence of cyclosporine A. Triton X-100 treatment results in the release of the total cytochrome *c* available in cells. (**c**) Quantification of cytochrome *c* released in cells treated with different concentrations of curcumin and inhibition of this release by EGTA-AM or BAPTA-AM, which control intracellular calcium levels after curcumin addition. (**d**) Electron microscopy picture of autophagic vacuoles in cells treated with 25 *μ*M curcumin for 24 h. AV, autophagic vacuoles; SM, swollen mitochondria; V, vacuole.

Although isolated mitochondria are sensitive to curcumin, it is unclear whether this is relevant to whole cells, because the amount of curcumin internalized may be insufficient to trigger PTP opening at the cellular level. Even at an external curcumin concentration of 25 *μ*M, the intracytoplasmic concentration was only 1.5 *μ*M and thus below the minimal concentration needed for PTP opening (≥2.5 *μ*M for isolated mitochondria; Figure 3d). To investigate this issue, we used electron microscopy to examine Huh-7 cells treated with 25 *μ*M curcumin for 1, 3 or 24 h. Swollen mitochondria were observed within 1–3 h of treatment (Figure 4a), and the swelling was even more pronounced at 24 h. In control samples, the diameter of the mitochondria was 0.8–1 *μ*m, whereas after 24 h of curcumin treatment it was 1.5–2 *μ*m. Treated cell mitochondria had a clear matrix and fewer cristae membranes than those in untreated cells. This was accompanied by a pronounced increase in the size of the endoplasmic reticulum (ER) lumen, which seemed to swell in parallel with mitochondria (Figure 4a). Mitochondrial swelling was accompanied by the release of cytochrome *c* into the cytoplasm (inhibited by cyclosporin A (CsA); Figure 4b), EGTA-AM (a slow calcium chelator) and BAPTA-AM (a fast calcium chelator; Figure 4c). This indicates that intracytoplasmic calcium is involved in mitochondrial swelling and that the calcium pool is accessible to chelation (Figures 4b and c).

Cells treated with 25 *μ*M curcumin exhibit autophagosomes (AV) containing digested mitochondria and cytosolic fragments (Figure 4d). These AV could be distinguished from classical vacuoles, which were also present in the cytoplasm but remained translucid.

### Intracellular curcumin is associated with the ER and a subpopulation of lysosomes

The effects of curcumin, including the drop in ΔΨ_m_ and the production of ROS, do not appear to be consequences of its direct action on mitochondria. Indeed, the very early release of calcium into the cytoplasm following curcumin treatment (Figures 2e and f) prompted us to investigate potential interactions between curcumin and the ER, which contains the main cellular pool of free calcium.

**Figure 5:**
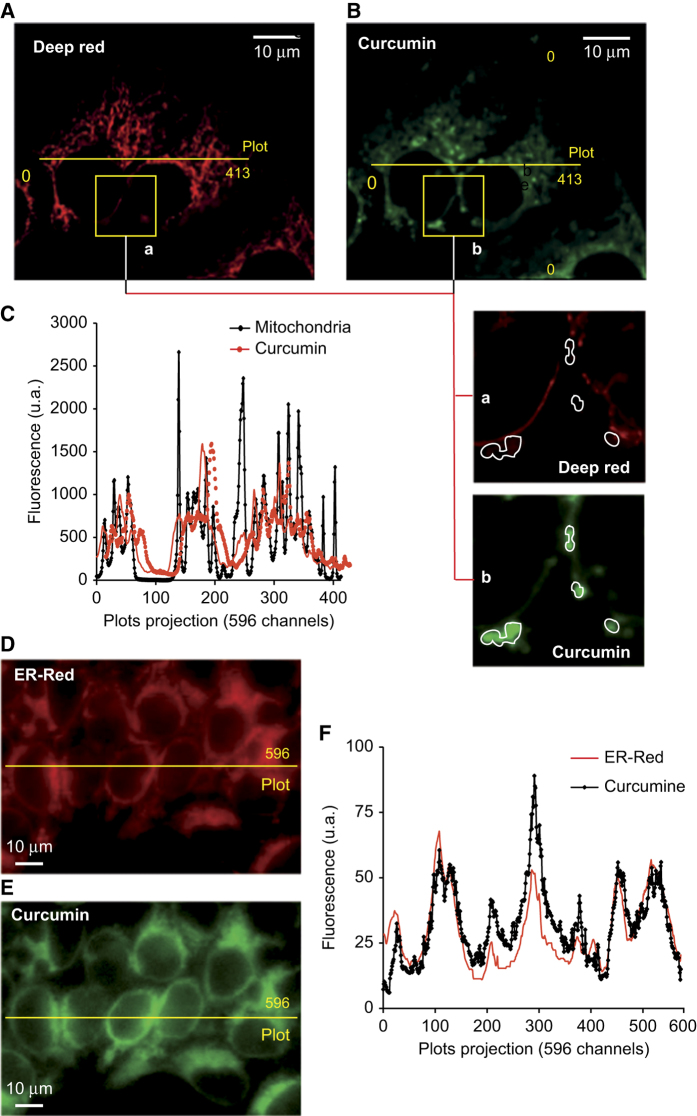
Confocal microscopy analysis of curcumin autofluorescence and mitochondrial staining with the cationic and lipophilic dye deep red. (**A**–**C**) The mitochondrial compartment (**A**) was stained with 40 nM deep red which fluoresces at 633 nm, whereas curcumin (**B**) fluoresces at 530±30 nm after excitation at 488 nm. (**A** and **B**) Panels (10 *μ*M scale) show enlarged portions of **A** and **B**, respectively. Curcumin spots (b) in green are surrounded by lines, which are clearly empty in (a) which shows the deep red staining of the mitochondrial compartment. (**C**) A plot of fluorescence along the yellow line shown in **A **and **B** consisting of 413 pixels. The main peaks corresponding to deep red staining do not co-localize with those corresponding to curcumin fluorescence. In **D**, the ER compartment was stained with ER tracker red and the picture was taken at 585±20 nm after excitation at 488 nm. (**E**) Curcumin fluorescence was measured at 530±30 nm after excitation at 488 nm. (**F**) A plot of fluorescence along the yellow line of 596 pixels shown in the picture; curcumin fluorescence is shown in black and ER-red tracker in red.

We therefore used confocal microscopy and the mitochondrial dye, deep red, to analyze simultaneously curcumin fluorescence and the mitochondrial compartment (Figures 5A–D). The mitochondrial network was clearly visible around nuclei and within the cytoplasm (Figure 5A), and this analysis confirmed the punctated pattern of curcumin fluorescence (Figure 5B). However, the curcumin signal did not colocalize with the mitochondria: curcumin fluorescent spots were close to, but did not overlap with, mitochondria (Figure 5C). Transected images confirmed that the distribution of curcumin and mitochondria was different (Figure 5C).

However, curcumin fluorescence colocalized perfectly with the ER, which was stained with ER red stain (ER-red; Figures 5D–F). We also compared curcumin fluorescence with the pattern of lysosome staining assessed with LysoTracker Red DN99 (Supplementary Figures S1a–c) some lysosomes colocalized with curcumin (Supplementary Figure S1c, stars*), whereas others did not (Supplementary Figure S1c, arrow). This was clearly visible in plots of transected sections of fluorescent images, on which the colocalization of lysosomes and curcumin are marked with stars (Supplementary Figure S1c).

### Curcumin induces autophagy followed by apoptosis

**Figure 6:**
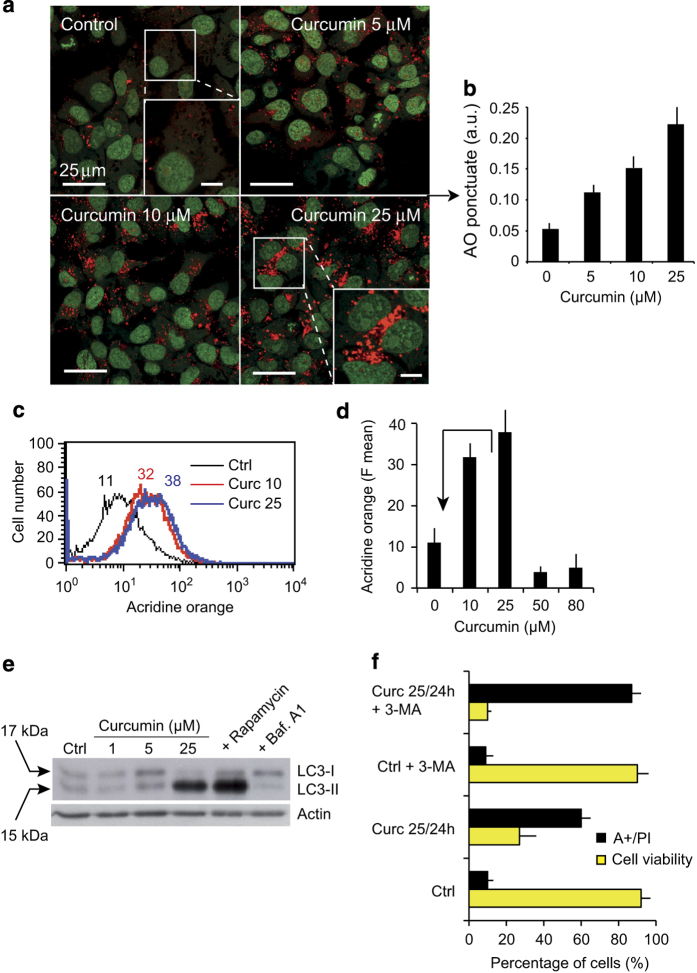
Curcumin induce autophagy. (**a**) Confocal microscopy of AO-stained vesicles in cells treated with different concentrations of curcumin (5, 10 or 25 *μ*M). In the section showing cells treated with 25 *μ*M curcumin, the enlarged panel (lower right panel) reveals the massive accumulation of AO-positive vesicles. (**b**) Quantification of AO-punctuate staining (intracytoplasmic vesicles). The means (±S.D.) of seven independent experiments are shown **P<0.01. (**c** and **d**) Flow cytometric analysis of AO red fluorescence. In **d**, the mean fluorescence values±S.D. of seven independent experiments is shown. (**e**) Western blot analysis of the conversion of LC3-I to LC3-II in cells treated with different curcumin concentrations (1, 5 or 25 *μ*M). Rapamycin was used to induce autophagy and Bafilomycin A1 to inhibit it. (**f**) Treatment with the autophagy inhibitor 3-MA promotes curcumin-induced apoptosis. Huh-7 cells were treated with curcumin (20 *μ*M) and/or 3-MA (10 mM) for 24 h. Cell viability was determined by the PI staining assay, and apoptosis evaluated with Annexin V-FITC. Positively-stained cells were counted using a FACScalibur 4C. Data are expressed as the mean±S.D., *n*=6. **P<0.01 *versus* control; *P<0.05 *versus* curcumin-treated group.

We observed that curcumin treatment leads to the formation of autophagic vacuoles containing degraded mitochondria (see above; Figure 4d). Given that dysfunctional mitochondria are usually subject to mitophagy, we examined the formation of autophagolysosomes by fluorescence microscopy following staining with the lysosomotropic agent acridine orange (AO).^19^ Control cells showed mostly green fluorescence with very little cytoplasmic red staining corresponding to lysosomes; however, curcumin-treated cells displayed many spots of red fluorescence, indicating the formation and accumulation of acidic vesicles (Figures 6a and b). At 25 *μ*M curcumin, AO red punctate staining was significantly higher than in control conditions (Figure 6b). Consistent with the findings of the confocal image analysis (Figures 6a and b), flow cytometry analysis showed that autophagosome formation increased with curcumin concentrations until 25 *μ*M (Figure 6d). However, at higher curcumin concentrations, that is ≥50 *μ*M, the numbers of AO-positive cells (Figure 6d) and the AO spots were lower (data not shown), suggesting that autophagy is inhibited and/or the vesicular system becomes more alkaline at these concentrations.

To examine further whether curcumin induces autophagy, we used western blotting to study the autophagy marker, LC3-II.^20^ Curcumin stimulated the time-dependent production of the microtubule-associated protein light chain 3 (LC3-I), and the accumulation of its processed form (LC3-II; Figure 6e). A high LC3-II/LC3-I ratio is a marker of autophagy. Quantification of LC3-II and actin in cells revealed that LC3-II levels were fivefold higher following 24 h of treatment with 25 *μ*M curcumin than in control conditions (Figure 6e). Huh-7 cells treated for 24 h with Rapamycin as a positive control showed high levels of LC3-II and those treated with Bafilomycin A1 as a negative control exhibited low levels of LC3-II (Figure 6e).

These findings indicate that curcumin may induce ER stress by promoting the formation of ubiquitinated misfolded proteins, leading to the activation of both autophagy and apoptosis. The autophagy inhibitor 3-methyladenine (3-MA) promotes the accumulation of ubiquitinated proteins in cells, thus exacerbating ER stress. We therefore used 3-MA to test whether ER stress has a role in curcumin-induced apoptosis in Huh-7 cells and whether the inhibition of autophagy stimulates curcumin-induced apoptosis by inducing ER stress. We treated cells with 3-MA and 25 *μ*M curcumin for 24 h (Figure 6f) and examined apoptosis by annexin V-staining and cell viability by PI staining. The inhibition of autophagy by 3-MA indeed stimulated apoptosis (Figure 6f).

### Curcumin-induced caspase activation

**Figure 7:**
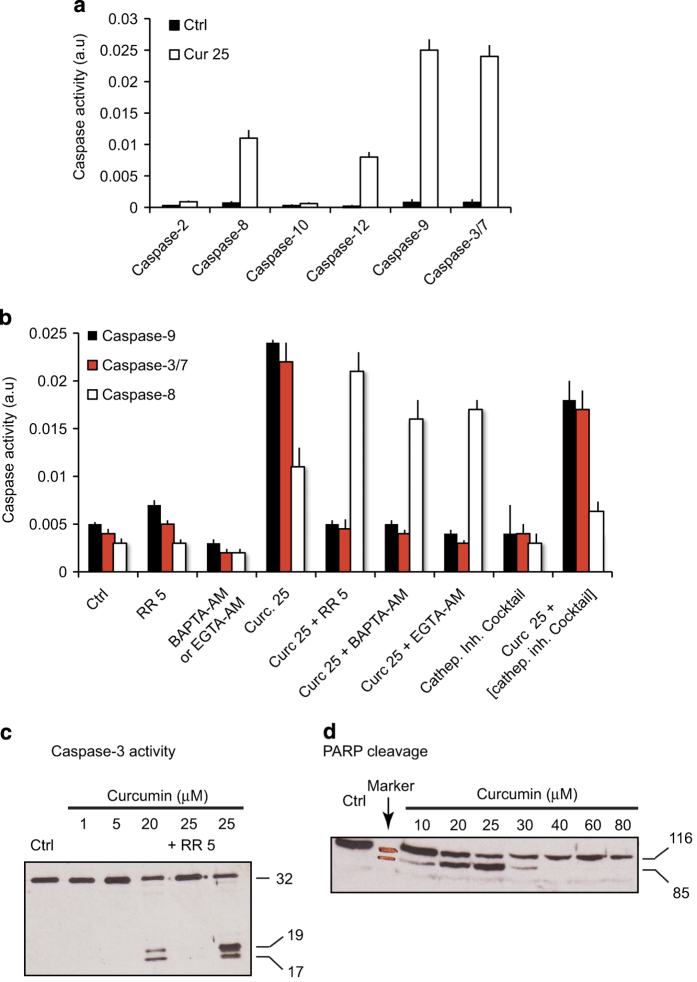
Caspase activity, regulation of caspase activity and PARP cleavage in curcumin-treated cells. (**a**) The activity of various caspases in cells treated with 25 *μ*M curcumin. (**b**) Regulation of caspase-3/7, -9 and -8 activity by calcium chelators (BAPTA-AM or EGTA-AM; 25 *μ*M), the mitochondrial calcium uniport inhibitor ruthenium red (5 *μ*M) and a cathepsin inhibitor cocktail in cells treated with 25 *μ*M curcumin for 24 h. (**c**) Western blot showing caspase-3 cleavage after treatment with 1, 5, 20 or 25 *μ*M curcumin for 24 h. One lane shows samples treated with 25 *μ*M curcumin after preincubation with 5 *μ*M of ruthenium red. (**d**) Western blot showing PARP cleavage in cells treated with various concentrations of curcumin (from 0 to 80 *μ*M).

We examined the activity of several caspases during curcumin-induced cell death (apoptosis and/or necrosis) (Figure 7a). Caspases-3/7, -9, -8 and -12 were all activated to different extents, and caspases-2 and -10 were not activated (Figure 7a). Given that the mitochondrial pathway is activated after destabilization of mitochondrial homeostasis and cytochrome *c* release (Figures 4b and c) caused by ER stress and high cytoplasmic calcium concentrations, it is not surprising that caspases-9 and -3/7 are activated following exposure to curcumin. Caspases-12 and -8 were activated to a lesser extent. Caspase-12 is activated in stress conditions (ROS production), although its precise function in this pathway is unclear. Caspase-8 activation may be related to lysosomal destabilization resulting from intracytoplasmic curcumin, which activates cathepsins. Indeed, cathespins activate caspase-8 and cleave Bid. Furthermore, Ca^2+^-dependent calpain activation may also stimulate the activity of caspase-8.

### Effect of calcium chelation or calpain/cathepsin inactivation on the activity of caspase-3/7, -9 and -8

We pretreated Huh-7 cells with ruthenium red (an inhibitor of the mitochondrial Ca^2+^ antiporter), BAPTA-AM (a fast Ca^2+^ chelator), EGTA-AM (the AM moiety increases its cellular permeation), a cocktail of calpain inhibitors (pepstatin+E64d) or a cocktail of cathepsin inhibitors for 1 h. We then treated the cells with 25 *μ*M curcumin for 24 h and analyzed the activity of caspase-9, -3/7 and -8 (Figure 7b). Ruthenium red, BAPTA-AM and EGTA-AM impaired the activity of caspase-9 and -3/7. Surprisingly, the inhibition of caspase-9 and -3/7 was associated with high caspase-8 activity (Figure 7b). Thus, inhibition of mitochondrial cell death appears to exacerbate the alternative lysosomal pathway. These findings confirm that calcium is involved in curcumin-induced mitochondrial destabilization and the activation of caspase-9 and -3/7 via cytochrome *c* release.

The cathepsin inhibitor cocktail had small effects on caspase-9 and -3/7 (Figure 7b), but clearly impaired caspase-8 activity, which implicates the cathepsin pathway in caspase-8 activation.

Next, we treated cells with 5 *μ*M ruthenium red for 30 min and then with curcumin (Figure 7b) and examined caspase-3 cleavage by western blotting. No caspase-3 cleavage was detected below 5 *μ*M curcumin, although 5 *μ*M curcumin was sufficient to induce calcium influx, a ΔΨ_m_ drop and ROS production. This concentration may be enough to initiate early autophagic processes but not apoptosis. However, caspase-3 cleavage was detected at 20 *μ*M curcumin but was stronger at 25 *μ*M. Cleavage was completely inhibited by 5 *μ*M ruthenium red.

We also tested whether proapoptotic signaling was intact by examining PARP cleavage (Figure 7c). PARP was efficiently cleaved at curcumin concentrations up to 25 *μ*M (which induce apoptosis, Figure 2a) whereas at higher concentrations, which induce mainly necrosis, PARP was degraded, as observed during cytotoxic death (Figure 7d).

### Curcumin-induced apoptosis is also mediated via cathepsin and the calcium-dependent activation of calpain

**Figure 8:**
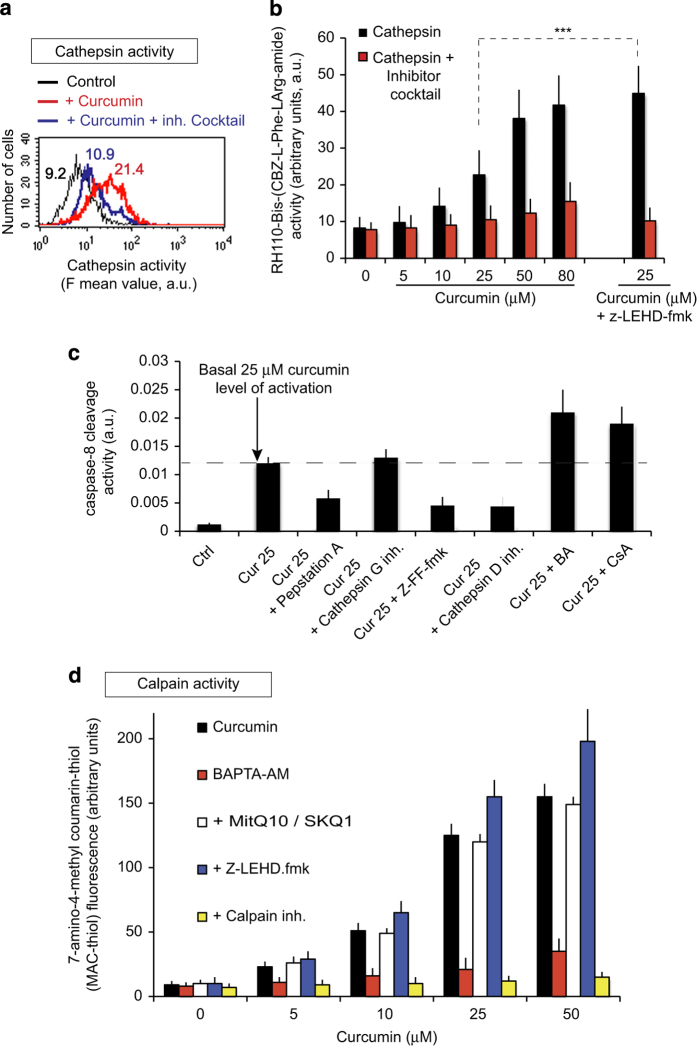
Cathepsin and calpain induction by curcumin. (**a**) Flow cytometry analysis of the cathepsin activity following the incubation of cells with 25 *μ*M curcumin for 24 h in the presence or absence of a cocktail of cathepsin inhibitors. (**b**) Cathepsin activity in Huh-7 cells treated with various concentrations of curcumin for 24 h. The effects of z-LEHD, an inhibitor of caspase-9, were tested in samples incubated with 25 *μ*M curcumin. (**c**) Caspase-8 activity in cells treated with 25 *μ*M curcumin and one of several cathepsin inhibitors (pepstatin A, cathepsin G inhibitor and Z-FF-fmk) or pore opening inhibitors (BA and CsA). (**d**) Curcumin-induced calpain activity in the presence of a calcium chelator, BAPTA-AM, a mitochondrially targeted antioxidant, an inhibitor of capase-9, Z-LEHDfmk, or a calpain inhibitor.

Since curcumin is taken up by a subpopulation of lysosomes (Supplementary Figures S1a and b), we examined cathepsin activity by analyzing the cleavage of Rh110-bis-CBZ-Lphe-l-Arg-amide using flow cytometry because lysosomal membrane permeabilization (LMP) is usually associated with cathepsin release (Figure 8a). Cathepsin activity was higher in curcumin-treated Huh-7 cells than in control cells and was inhibited by a cathepsin inhibitor cocktail (Figure 8a). Cathepsin activity increased with increasing curcumin concentrations (Figure 8b). We also tested whether cathepsin activity was affected by inhibition of the mitochondrial pathway. In cells treated with 25 *μ*M curcumin, cathepsin activity was significantly higher in than presence of the caspase-9 inhibitor Z-LEHD-fmk than in control conditions (*P*<0.05; Figure 8b).

We used several cathepsin inhibitors to modulate caspase-8 activity (Figure 8c). Pepstatin A, Z-FF-fmk and the cathepsin D inhibitor efficiently abolished cathepsin activities and therefore inhibited caspase-8 activity whereas the cathepsin G inhibitor did not (Figure 8c). Given that the caspase-9 inhibitor Z-LEHD promoted caspase-8 activity, we tested whether inhibition of PTP with either CsA or bongkrekic acid (BA) had the same effect. Both CSA and BA promoted caspase-8 activity, probably because they block mitochondrial events and cytochrome *c* release.

Given that curcumin induces mitochondrial swelling through a calcium-dependent mechanism (Figures 2e and f), we investigated whether the calcium-dependent activation of calpain is involved in this process (Figure 8d). Calpains were activated by curcumin and this effect was inhibited by calpain inhibitors. Next, we studied whether caspase inhibition influenced calpain activation and whether the ROS generated by mitochondria interfered with calpain activity. The caspase-9 inhibitor Z-LEHD-fmk stimulated slightly calpain activity, whereas MitoQ10 or SKQ1, which efficiently inhibit mitochondrial ROS production (Supplementary Figure S2) through mitochondrial dysfunction and PTP opening, did not interfere with calpain activity. By contrast, Ca^2+^ chelation by BAPTA-AM inhibited calpain activity (Figure 8d).

### Antioxidants and modulation of curcumin-induced cell death

We then investigated the effects of various antioxidants on curcumin-induced events. We used three antioxidants targeted to the cytoplasm, NAC, Vitamin E and Trolox, and two antioxidant targeted to mitochondria (mitoQ10 or SKQ1). Each of the three cytoplasmically targeted antioxidants almost completely abolished curcumin-induced ROS production (Supplementary Figure S2 and Table 1). However, MitoQ10 and SKQ1, mitochondrially targeted antioxidants, were the most efficient on curcumin-induced ROS production.

## Discussion

### Uptake and localization of curcumin

Many studies have investigated the use of curcumin for therapeutic purposes, although few have quantified curcumin uptake or analyzed its intracellular localization. Descriptions of the intracellular localization of curcumin are poor^21^ and in some cases, erroneous. Here, found that curcumin is rapidly taken up by cells (Figure 1a, upper panel) in a 1/20 ratio if referred to the external concentration. Curcumin is very lipophillic, so it may accumulate at intracellular membranes, with deleterious effects.^22^ Nevertheless, our findings conflict with previous reports showing that curcumin accumulates at the plasma membrane and in the nucleus.^21^

We detected curcumin at the ER (Figures 5D and E) and lysosomes (Supplementary Figures S1a–c) level. Costaining experiments confirmed that the primary target of curcumin is not the mitochondrial compartment. A further analysis of curcumin-induced apoptosis indicated that mitochondrial destabilization was an early event, which manifests as a ΔΨ_m_ drop with ROS production. The calcium increased is almost in parallel with curcumin accumulation, and preceded ROS production and the ΔΨ_m_ decrease (Figures 2e and f).

### PTP opening and the induction of cell death by curcumin

Experiments with isolated purified mitochondria demonstrated that the PTP opens at curcumin concentrations above 2.5 *μ*M (Figure 3d). Morin *et al.* and Ligeret *et al.* ^23^ report PTP opening at high concentrations of curcumin. However, it seems unlikely that this PTP opening is caused by the direct action of curcumin on mitochondria *in vivo*, except at very high doses (30 *μ*M) at which curcumin is toxic and induces necrotic-like cell death. Confocal microscopy (Figure 4) of cells stained with curcumin and a mitochondrial marker demonstrated that curcumin does not accumulate in mitochondria but is present nearby (Figures 5A–C). Thus, PTP opening is not the result of a direct interaction between internalized curcumin and mitochondria. EM pictures of Huh-7 cells revealed swollen mitochondria after 1 h of treatment with 25 *μ*M curcumin, and big, swollen, translucent mitochondria with few cristae membranes after 24 h (Figure 4a). These swollen mitochondria were nearby huge empty vacuoles or autophagic vacuoles, and others were inside autophagosomes (mitophagy; Figure 4d).

### Curcumin destabilizes the ER and promotes calcium release

Confocal microscopy revealed that curcumin accumulates at the ER membrane (Figures 5D–F). Electron microscopy showed a swollen appearance of the ER that is probably caused by intracellular ion imbalance eventually leading to osmotic lysis. Indeed, curcumin activates the unfolded protein response that leads to the the upregulation of C/EBP homologous protein and GADD34, the principal mediators of proapoptotic unfolded protein response.^24^ Curcumin induces the classical ER stress pathway accompanied by increased expression of GP78 and C/EBP homologous protein.^25^ Curcumin-mediated ER stress involves the sarcoplasmic/endoplasmic reticulum Ca^2+^-ATPase2.^25^ Effectively, curcumin-induced ER stress provoke the release of calcium, which is rapidly taken up by the mitochondrial via the mitochondria calcium uniporter. The cytochrome *c* was released from swollen mitochondria, but CsA, BA, inhibitors of PTP opening, and the intracellular calcium chelators EGTA-AM and BAPTA-AM, prevented this process (Figures 4b and c). This implicates calcium in curcumin-induced PTP opening that leads to cytochrome *c* release and subsequent caspase activation (Figure 7b).

### Curcumin colocalizes with a subpopulation of lysosomes and induces lysosomal membrane permeabilization

Various death stimuli can induce this lysosomal pathway which is characterized by permeabilization and, possibly, partial rupture of the lysosomal membrane with the subsequent release of the intra-lysosomal content into the cytoplasm, including cathepsins,^26^ calpains^27^ and chemotrypsin.^28,29^ Some internalized curcumin localizes in the lysosomal compartment (Supplementary Figure S1) where it induces a LMP content into the cytoplasm and activation of cathepsin (Figures 8a–c). When released into the cytosol, lysosomal proteases, in particular cathepsins B and L and aspartate cathespsin, can contribute to apoptotic processes either themselves or by acting together with caspases.^26,30^ LMP may therefore be an early event of apoptosis.^31,32^ We report that curcumin affects the lysosomal compartment. Curcumin treatment promoted the accumulation of AO into the cytosol.^33^ Bafilomycin A1, a specific inhibitor of the lysosomal proton pump, completely abolished curcumin-induced AO red staining and thus protected lysosomes from permeabilization (Figure 6e). Abnormally high LMP has been reported in various types of tumor cells exposed to putative anti-cancer chemopreventive agents during apoptosis.^34,35^ One report in particular suggests that curcumin induces LMP.^35^

Curcumin may simply insert itself into the membrane bilayer, like cholesterol,^36^ thus enhancing LMP. This may be associated with the release of calcium from the ER which may act synergistically with ROS: calcium activates calpains, which contribute to the disruption of the lysosomal membrane^37^ and the formation of macroautophagosome,^38,39^ and ROS destabilizes the lysosomal membrane (Figure 8d). Indeed, curcumin-induced LMP activated cathepsin L and D (Figure 8c). These observations are consistent with recent studies showing that the lysosomal translocation of cathepsin D in human retinoblastoma Y79 cells promotes mitochondrial membrane permeability thus resulting in apoptosis.^40^ Furthermore, cathepsin B is abnormally active and results in similar events in pancreatic cancer cells.^41^ We show that curcumin significantly stimulates cathepsin activity, which may affect the cleavage of Bid and the subsequent delocalization of Bax to the mitochondrial membrane followed by caspase-3 activation. At curcumin concentrations ≥50 *μ*M, cathepsin activity was high although this was probably not the result of a deregulated signaling pathway but the indication that cathepsins actively participate in the dismantling of the cell during necrosis.

However, in our system, where the ER stress is a nearly event, it is possible that calcium release from the ER increases mitochondrial dysfunction, ROS production and cytochrome *c* release. ROS originating from destabilization of the electron transport chain help to destabilize the lysosomal compartment via peroxidation of membrane lipids. The NPe6 photosensitizer, when located in lysosomal membranes,^42^ generates singlet oxygen upon photoactivation, leading to the fast release of cathepsins into the cytosol. Furthermore, apoptosis is initiated by UVA radiation and by H_2_O_2_, both of which trigger cell death mainly by generating oxidative stress associated with LMP. Other inducers of apoptosis, like vacuolar ATPase inhibitors^43^ and *N*-(4-hydroxyphenyl) retinamide,^44^ cause ROS-dependent LMP. One or more of these various mechanisms may be involved in curcumin-induced lysosomal destabilization.

Our findings clearly confirm the importance of lysosomes in apoptotic processes: curcumin accumulated rapidly in lysosomes (Supplementary Figure S1) and ROS production was an early event inhibited by ROS scavengers like NAC, MnBTAP and more efficiently by mitochondrially targeted MitoQ10 or SKQ1. ROS inhibition by NAC significantly delayed curcumin-induced apoptosis.^45^

### Autophagy is an early event during curcumin-induced cell death

Autophagy is an evolutionarily conserved, multistep lysosomal degradation process distinct from apoptosis in which a cell destroys long-lived proteins and damaged organelles. Autophagy is important for cell survival, and the inhibition of autophagy promotes cell death. However, autophagy can also accompany cell death. Curcumin treatment promotes autophagy before signs of cell death manifest, as evidenced by images of autophagosome formation, AO staining (Figures 6a–c) and LC3II formation assessed by western blotting (Figure 6e). Our results are consistent with the recent publication showing that autophagy is involved when the curcumin derivative bis-dehydroxycurcumin is used on colon cancer cells.^46^ Evidence now exists of complex interplay between autophagy and apoptosis. Notably, many genes, such as p53 and Bcl-2 family members, are shared by these two pathways.

The accumulation of poly-ubiquitinated proteins and the induction of ER stress promote autophagy,^47^ which in turn may trigger mitochondrial-dependent apoptosis. Pharmacological and RNAi-mediated inhibition of ER-stress and autophagy has revealed that autophagy potentiates the anti-proliferative effect of curcumin.^25^ Our findings demonstrate that curcumin is a pro-autophagic cytotoxic drug, providing further evidence of its therapeutic potential against developing cancer cells.

In conclusion, we report for the first time that the ER and the lysosomal compartment act in synergy to induce apoptosis and cell death in curcumin-treated Huh-7 cells. Moreover, ER stress is also involved in the establishment of mitochondrially linked autophagic processes that precede apoptosis and necrosis. These early events were almost completely blocked by the calcium chelators EGTA-AM and BAPTA-AM, and inhibitors of the mitochondrially generated ROS species, that is, MitoQ10 and SKQ1 (Supplementary Figure 2 and Table 1). Curcumin destabilizes the ER leading to calcium release into the cytosol, which links primary ER-stress-related events with classical mitochondrially induced apoptosis. We also found that curcumin triggers the release of lysosomal cathepsins into the cytosol. These events are blocked by NAC, which shows that lysosomal destabilization is linked to ROS production, although the accumulation/insertion of curcumin in the lysosomal membrane *per se* may explain LMP and the activation of cathepsin/chemotrypsin.

Apoptosis/necrosis were markedly impaired by pre-treatment of cells with NAC or broad spectrum caspase inhibitors. The increase in MMP was partly prevented by cathepsin inhibitors, suggesting that lysosomal destabilization is upstream from mitochondrial destabilization.

We propose that curcumin mediates cell apoptosis through lysosomal destabilization. Upon entry into the cell, curcumin destabilizes the lysosomal membrane, triggering the release of lysosomal enzymes into the cytosol, which in turn induces MMP via Bid cleavage and Bax delocalization, resulting in mitochondrial swelling, cytochrome *c* release and ROS production. ROS then further destabilize lysosomal membrane integrity, thus enhancing LMP in curcumin-treated cells.

**Figure 9:**
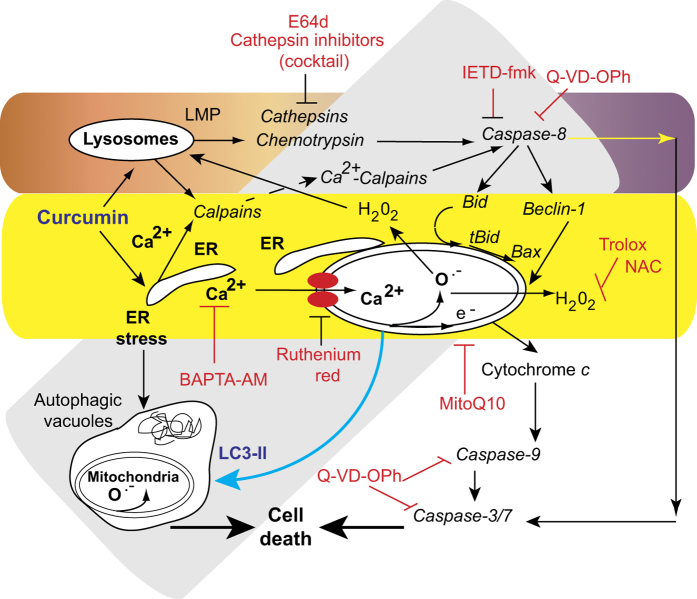
Schematic representation of curcumin effects. Curcumin mainly targets the ER and lysosomes but to a different extent. The classic apoptotic pathway is mediated by calcium release from the ER. Uptake of this calcium by mitochondria disrupts mitochondrial homeostasis. Calcium alters mitochondrial electron transport causing substantial ROS production (both superoxide anions and hydrogen peroxide), which leads to the opening of the permeability transition pore in the mitochondrial membrane. Consequently, cytochrome *c* is released and the caspase-9 and -3/7 pathway is activated leading to cell death (mostly by apoptosis). Furthermore, the ER stress pathway leads to the formation of autophagic vacuoles that attempt to eliminate the dysfunctional mitochondria. The cleavage of Beclin-1 is associated with apoptosis and leads to the accumulation of autophagic vacuoles. Despite the activation of autophagy, cells undergo necrotic cell death following these initial apoptotic events. A lysosomal pathway may also be active. Curcumin destabilizes lysosomal membranes leading to lysosomal membrane permeability and the activation of both cathepsins and chemotrypsins. Activated caspase-8 leads to Beclin-1 cleavage. The increase in cytosolic calcium concentrations activates calpains that contribute to the degradation process and accelerate cell death. The various inhibitors used in this work are indicated with the pathways they affect. Curcumin interacts with the ER and lysosomes.

These events are summarized in Figure 9. Curcumin inserts into the ER and lysosomal membrane. Calcium is rapidly released as a result of ER stress, and strongly promotes mitochondrial destabilization, involving the production of superoxide anions and H_2_O_2_, mitochondrial membrane alterations and bioenergetic changes. These events promote MMP and cytochrome *c* release followed by the activation of executive caspases. However, at low curcumin concentrations and early during this sequence of events, curcumin induces autophagic ‘survival’ processes, which attempt to discard nonfunctional mitochondria and save cells from further destruction. However, if the initial crosstalk between apoptosis and autophagy is unsuccessful, a third pathway leads to cell death. In this case, LMP results in the release of cathepsins which activate caspase-8 and -3/9, to guarantee the destruction of cells.

Our findings suggest that an intricate crosstalk between apoptosis and autophagy determines the overall fate of the cell. Indeed, the final outcome of autophagy depends on: (i) the stress-inducing stimulus and (ii) the cellular context. Indeed, autophagy can help cells undergoing ER stress to survive, by eliminating unfolded proteins, or it can lead to cell death.^46,48^ Taken together these results imply a fail-secure mechanism regulated by autophagy in the action of curcumin, suggesting a therapeutic potential for curcumin. Then offering a novel and effective strategy for the treatment of malignant cells.

## Materials and Methods

### Chemicals and reagents

Culture medium RPMI-1640, fetal bovine serum, penicillin–streptomycin and l-glutamine were purchased from GIBCO BRL (Invitrogen, Grand Island, NY, USA). BA (10 *μ*M), CsA (5 *μ*M), curcumin (IUPAC name, (1E,6E)-1,7-bis-(4-hydroxy-3-methoxyphenyl)-1,6-heptadiene-3,5-dione; CAS number: 458-37-7), vitamin E, NAC, mClCCP (carbonyl cyanide m-chlorophenylhydrazone), ter-butylhydroperoxide (5 *μ*M), TPP^+^ (tetraphenylphosphonium chloride), Triton X-100 and calpain inhibitor I (1 *μ*M, *N*-acetyl-Leu-Leu-norleucinal, Ac-LLnL-CHO) were from Sigma-Aldrich Chemical Co. (St. Louis, MO, USA). Calpain inihbitor II (1 *μ*M, Ac-LLM-CHO, *N*-Acetyl-Leu-Leu-Methional) was from BioVision, Mountain View, CA, USA. Trolox is Hoffman-LaRoche's trade name for 6-hydroxy-2,5,7,8-tetramethylchroman-2-carboxylic acid, a water-soluble derivative of vitamin E. AO, 7-dichlorodihydrofluorescein diacetate, hydroethine, 3,3’-dihexyloxacarbocyanine iodide, PI and Fluo-4/AM were purchased from Molecular Probes, Eugene, OR, USA (Invitrogen, Eugene, OR, USA). The inhibitors used were cathepsin B inhibitor (CA074-Me, 50 *μ*M, Sigma-Aldrich SARL, Saint-Quentin Fallavier, France), cathepsin G inhibitor 1 (10 *μ*M, Calbiochem, Merck Chimie SAS, Fontenay sous Bois, France), cathepsin L inhibitor (10 *μ*M Z-FF-FMK, Calbiochem, Merck Chimie SAS), and cathepsin D inhibitor (10 *μ*M, pepstatin A, Sigma-Aldrich SARL) dissolved, in most cases, in DMSO (concentration±0.05%). In addition, 3-(4-Iodophenyl)-2-mercapto-(Z)-2-propenoic acid (PD 150606), a cell-permeable noncompetitive inhibitor of calpains 1 and 2, was provided by Calbiochem (Merck Chimie SAS). Mito Q10 was used at 5 *μ*M and was a gift from Dr. Murphy (Medical Research Council Dunn Human Nutrition Unit, Cambridge, UK). SkQ1 (plastoquinonyl decyltriphenyl phosphonium bromide) was a gift from V.P. Skulachev (Lomonosov Moscow State University, Moscow, Russia), EGTA-AM (cell permeable) and BAPTA-AM (cell permeable) were from Life Technology, Invitrogen. BAPTA-AM is a cell-permeable chelator, which is highly selective for Ca^2+^ over Mg^2+^, and can be used to control intracellular levels of Ca^2+^. BAPTA-AM is more selective than EDTA and EGTA for Ca^2+^, and its binding is also less sensitive to pH, which is important because the pH may change during early autophagy and apoptosis.

### Cells

Human hepatoma-derived Huh-7 cells were obtained from the RIKEN BioResource Center, Tsukuba, Japan, and were grown in the presence of 5% CO_2_ in Dulbecco’s modified Eagle’s medium containing high glucose 4.5 g/l (25 mM, Sigma-Aldrich, St. Louis, MI, USA) with 5% fetal bovine serum (Hyclone, Logan, UT, USA).

### Confocal microscopy

Cells were seeded onto glass coverslips at a concentration of ~5×10^5^ cells per well in six-well plates. Cells were treated with 25 *μ*M curcumin for 24 h in Dulbecco’s modified Eagle’s medium without phenol red and supplemented with 3% charcoal-treated calf serum. For immunofluorescence, all steps were carried out at room temperature. The coverslips were washed twice in 1× phosphate-buffered saline (PBS). Mounted cells were observed and images were recorded using a Zeiss LSM 510 confocal microscope (Carl Zeiss Meditec France SAS, Le Pecq, France) with a 40X Plan-Neofluar 1.3 numerical aperture oil objective and LSM Image Browser, or on a Nikon Eclipse TE-2000 E microscope (Nikon France S.A, Champigny sur Marne, France). Deconvolution and three-dimensional reconstitution were performed with Autoquant imaging Autodeblur version X 1.4.1 (AutoDebur and Autovisualize; Mediacybernetics, Bethesda, MD, USA). ImageJ 1.37v software was used for images analysis (imagej.nih.gov/ij). 

### Determination of ΔΨ_m_, ROS and cytosolic Ca^2+^ levels

Huh-7 cells at a density of 2×10^6^ in six-well plates were treated with 0–100 *μ*M of curcumin for 0 to 48 h as indicated. After treatment, cells were trypsinized, harvested, washed and resuspended, with their supernatant in calcium-free PBS (pH 7.2). To determine ΔΨ_m_, tetramethyl rosamine methyl ester or 3,3’-dihexyloxacarbocyanine iodide were added to a final concentration of 40 nM. To determine superoxide anion and H_2_O_2_ concentrations, 1 *μ*M hydroethidine and 5 *μ*M 2′,7′-dichlorodihydrofluorescein diacetate were used, respectively. The following antioxidants were used: a mitochondria-targeted antioxidant, Mito-Q_10_ (500 nM; the coenzyme-Q analog attached to a triphenylphosphonium cation targets the antioxidant to the mitochondrial matrix), SKQ1 (plastoquinonyl-decyl-triphenylphosphonium), Vitamin E (1 mM) and 6-hydroxy-2,5,7,8-tetramethylchroman-2-carboxylic acid (Trolox; 1 mM), an artificial vitamin E analog and NAC (5 mM). We tested the effects of ROS scavengers (NAC, Trolox, MitoQ10 and SkQ1) on Huh-7 cells treated with curcumin. Huh-7 cells were plated at ~2×10^6^ cells/well in six-well plates and were incubated for 3 h with or without antioxidants. Curcumin (25 μM) was then added and cell viability and ΔΨ_m_ were determined. Fluo3-AM (stock solution: 3 mg/ml) was used to determine cytosolic concentrations of calcium. EGTA-AM was added to cell cultures at 25 *μ*M and the samples maintained in the dark for 30 min at 37 °C before measurements.

Double staining was used to examine cell viability involving propidium iodide (PI; 1 mg/ml) with 3,3’-dihexyloxacarbocyanine iodide, dichlorofluorescein diacetate and Fluo4-AM, or TO-PRO-3 iodide (1 mg/ml) with hydroethine and tetramethyl rosamine methyl ester. Double staining with YO-PRO-1/PI (Molecular Probes) and Annexin-V/PI staining was also used to distinguish between viable, apoptotic and necrotic cells. Annnexin-V FITC (Immunotech, Beckman-Coulter, Beckman Coulter International S.A., Nyon, Switzerland) was used to detect the aberrant exposure of phosphatidyl serine residues on the outer surface of the plasma membrane. All samples were analyzed by flow cytometry as described previously^49,50^ on a FACS Calibur 4C. Forward scatter (FSC) and side (90° angle) scatter (SSC) were measured after excitation at 488 nm. The values reported are the mean fluorescent values and the standard deviations are calculated on a linear scale with the Cellquest program.

### Determination of NAD(P)H autofluorescence

NAD(P)H fluorescence was measured under illumination with a multiline ultraviolet light at 400 mW; the light emitted was detected with a 424±40 nm bandpass to select NAD(P)H fluorescence.^50^ Changes in the auto-fluorescence of control and curcumin-treated cells were recorded.

### Determination of caspase-3, -8 and -9 activity by flow cytometry

Huh-7 cells were plated onto 6-well plates at a density of 2×10^6^ cells/well and incubated with 25 *μ*M curcumin for various periods of time and harvested. The samples were then washed, re-suspended in 50 *μ*l of 10 *μ*M substrate solutions (PhiPhiLuxG1D2 for caspase-3, CaspasLux8 for caspase-8 and CaspasLux9 for caspase-9) and incubated at 37˚C for 1 h, according to the manufacturer’s (OncoImmune, Inc., Rockville, MD, USA) instructions. The cells were washed again with PBS and analyzed by flow cytometry.

### Caspase activation, fluorimetric assays

Isolated Huh-7 cells were washed and suspended in calcium-free buffer solution (140 mM NaCl, 1.13 mM MgCl_2_, 4.7 mM KCl, 10 mM glucose, 0.1 M EDTA, and 10 mM HEPES, pH 7.2). Cells were then loaded at room temperature for 30 min with fluorescent indicator-linked substrates for activated caspase-2 (10 *μ*M Z-VDAD-R110; Molecular Probes), caspase-8 (10 *μ*M Z-IETD-R110; Molecular Probes), caspase-9 (10 *μ*M Z-LEHD-R110; Molecular Probes), or general caspases (10 *μ*M R110-aspartic acid amide; Molecular Probes) or loaded at 37 °C for 1 h with substrates for caspase-10 (50 *μ*M AEVD AFC; BioVision) or caspase-12 (50 *μ*M ATAD-AFC; BioVision).

### Acridine orange staining for autophagy

Huh-7 cells were treated with various concentrations of curcumin or DMSO (control) for 24 h and then incubated with 2 *μ*M AO for 15 min at 37 °C. The samples were examined under a Zeiss AxioSkop 40 fluorescence microscope (Carl Zeiss, Iena, Germany) or analyzed by flow cytometry. AO preferentially accumulates in acidic vesicles and generates red fluorescence upon excitation at 488 nm. However, AO shows green fluorescence in organelles at basic and neutral pH or when intercalated in DNA. This experiment needs to be validated by other classical methods to determine autophagy, that is, electron microscopy and LC3 cleavage.

### Analysis of cathepsin activity by flow cytometry

Cathepsin activity in live cells using 7-aminomethylcoumarin (AMC) substrates. Respective magic red (MR) substrates (Immunochemistry Technologies, LLC, Bloomington, MN, USA) for cathepsins B, L and S were Z-Arg-Arg-MR-Arg-Arg, Z-Phe-Arg-MR-Arg-Phe and Z-Val-Val-Arg-MR-Arg, respectively. General cysteine cathepsin inhibitor was E-64d (Peptide International, Louisville, KY, USA), and selective inhibitors for cathepsins L and S were Z-Phe-Phe-CHN2 and Z-Val-Val-Nle-CHN2, respectively (Bachem Bioscience, King of Prussia, PA, USA). Respective AMC substrates (Bachem Bioscience) for cathepsins B, L and S were Z-Arg-Arg-AMC, Z-Phe-Arg-AMC and Z-Val-Val-Arg-AMC. Stock solutions of substrates and inhibitor cocktails in DMSO were stored at −80 °C including [(Z—Arg)2Rh110, 2HCl], E64d, pepstatin and selective inhibitors of cathepsins L (Z-Phe- Phe-CHN2) and S (Z-Val-Val-Nle-CHN2). Cells were incubated with or without a cathepsin inhibitor cocktail under the conditions described above. Cells were then washed and resuspended in PBS. Cathepsin activity was measured by cleavage of AMC-selective cathepsin B, L or S substrate for 1 h at 37 °C. The fluorescence of liberated AMC was analyzed at ex 380 nm and em 435 nm in a spectrofluorometer. Cells incubated with the corresponding concentration of DMSO but without a cathepsin substrate were used as a background control. Percent inhibition was calculated as (1−(fluorescence units with inhibitor/ fluorescence units without inhibitor))×100%.

### Analysis of calpain activity by flow cytometry

7-Amino-4-chloromethyl coumarin, t-BOC-leucine-methionine amide (BOC-LM-CMAC) was purchased from Invitrogen. BOC-LM-CMAC is a calpain substrate which is well retained in live cells and its spectral properties differ from those of its product. The nonfluorescent-BOC-l-leucyl-methionine amide diffuses freely into cells but becomes unable to cross membranes after being conjugated to a thiol. Cleavage of t-BOC-thiol by calpain results in the release of fluorescent 7-amino-4-methylcoumarin-thiol (MAC-thiol). Cells were incubated tBOC-l-leucyl-methionine amide (10 *μ*M) for 30 min at 37 °C, trypsinized and the fluorescence at 405±30 nm of aliquots of 10 000 cells was analyzed with a FACS Aria II with excitation at 367 nm. PD 150606, a cell-permeable non-competitive inhibitor of calpains 1 and 2 was used to inhibit calpain activity.

### Purification of mouse liver mitochondria: respiratory activity, membrane potential and cytochrome *c* release

Three- to six-week-old mice (C57/Bl6) were killed by cervical dislocation. Mitochondria were isolated from livers in medium containing 0.3 M sucrose, 5 mM TES (*N*-tris[Hydroxymethyl]-methyl-2-aminoethanesulfonic acid), 0.2 mM EGTA and 0.1% BSA (wt/vol; pH=7.2). Mitochondria were prepared and purified on a Percoll gradient as described previously.^51^ Oxygen uptake and transmembrane potential (DY_m_) were monitored simultaneously in an oxygen electrode chamber (Hansatech Instruments Ltd, Pentney, Norfolk, UK). A Clark-type electrode was used to detect oxygen uptake and a tetraphenylphosphonium (TPP^+^)-sensitive electrode to determine membrane potential as described previously.^52^ For cytochrome *c *release has been realized as described by Gonzalvez *et al.* ^53^

### Permeability transition measurement

Mitochondrial swelling was estimated from the decrease in absorbance at 520 nm measured in a Uvikon 930 spectrophotometer (Kontron Instruments, Kontron AG, Augsburg, Germany). The medium contained 250 mM sucrose, 20 mM Tris-MOPS, the measurements optimal succinate transport activity and thus optimal succinate dehydrogenase activity. EGTA was included at a low concentration (10 *μ*M) to optimize experimental reproducibility. CsA was used at 2 *μ*M, and calcium was added to a final concentration of 25 *μ*M.

### Electron microscopy

Huh-7 cells were fixed by incubation with 1.25% glutaraldehyde in 0.1 M cacodylate buffer, pH 7.4 for at least 30 min at 4 °C. The specimens were then treated and analyzed as described previously.^54^

### Western blots

Cells were lysed in modified Laemmli's buffer (60 mM Tris (pH 6.8), 10% (vol/vol) glycerol, 2% (wt/vol) SDS and Bromophenol Blue, without 2-mercaptoethanol) by sonication for 30 s on ice and were then centrifuged at 3000×*g* for 5 min. The supernatants were boiled for 5 min at 100 °C and frozen at −80 °C. Protein concentration was determined by the micro-BCA protein assay (Pierce, Rockford, IL, USA). Cell lysates (20 *μ*g per lane) were resolved by SDS-PAGE (7.5% or 15% (wt/vol) polyacrylamide). Proteins were then electroblotted onto 0.45 *μ*m pore-size nitrocellulose filters, and the filters were blocked by incubation with 5% (wt/vol) non-fat milk in PBS containing 0.1% Tween-20 for 1 h and the filters were blocked by incubation with 5% (wt/vol) non-fat milk in PBS containing 0.1% Tween-20 for 1 h. The filters were then incubated for 1 h at room temperature with 1 *μ*g/ml anti-caspase 3 (polyclonal rabbit anti-caspase 3 serum; Pharmingen, San Diego, CA, USA) or monoclonal antibody C2.10 specific for poly(ADP-ribose) polymerase (1 *μ*g/ml) (purchased from Dr G.G. Poirier, Wellcome CRC Institute of Cancer and Developmental Biology, Cambridge, UK). To determine LC3 cleavage, rabbit anti-LC3-B, ref. L7543 from Sigma-Aldrich was used at 1 : 1000 and detected with anti-rabbit, RPN2124 from GE Healthcare (Veélizy-Villacoublay, France) at a dilution of 1 : 30 000. Blots were washed three times for 10 min with 0.2% Tween 20 in PBS, then incubated for 1 h with peroxidase-labeled anti-mouse or anti-rabbit immunoglobulins (at 1 : 5000 dilution). Blots were developed using an enhanced chemiluminescence detection system (ECL2; Amersham, UK).

## Figures and Tables

**Table 1:** Regulation of the curcumin-induced production of hydrogen peroxide by various antioxidants

**Conditions**	**DCF fluorescence (a.u.)**	
	**Control**	**+Curcumin 25 *μ*M**
No addition	8±3^a^	48±5
+NAC (5 mM)	5±4	21±8
+Trolox (1 mM)	6±5	17±6
+Vitamin E (1 mM)	7±4	18±6
+MitoQ10 (500 nM)	1±4	11±7
+SKQ1 (25 nM)	1±3	10±6

Abbreviation: DCF, dichlorofluorescein.

Huh-7 cells where incubated for 24 h with 25 *μ*M curcumin and then stained for 30 min at 37 °C with dichlorofluorescein diacetate. The resultant dichlorofluorescein fluorescence is expressed as the percentage of fluorescent cells that are more fluorescence or less fluorescent than control cells.

a Corresponds to basal ROS detection (i.e., control cells).

## Notes

Supplementary Figrue S1

Supplementary Figrue S2

Supplementary Figrue S3

Supplementary Figrue S4
