# Supplementary figures and images for: Curcumin induces crosstalk between autophagy and apoptosis mediated by calcium release from the endoplasmic reticulum, lysosomal destabilization and mitochondrial events
Source: Cell Death Discov. 2015 Oct 26;1:15017–. doi: 10.1038/cddiscovery.2015.17 (PMC4979459; doi:10.1038/cddiscovery.2015.17)

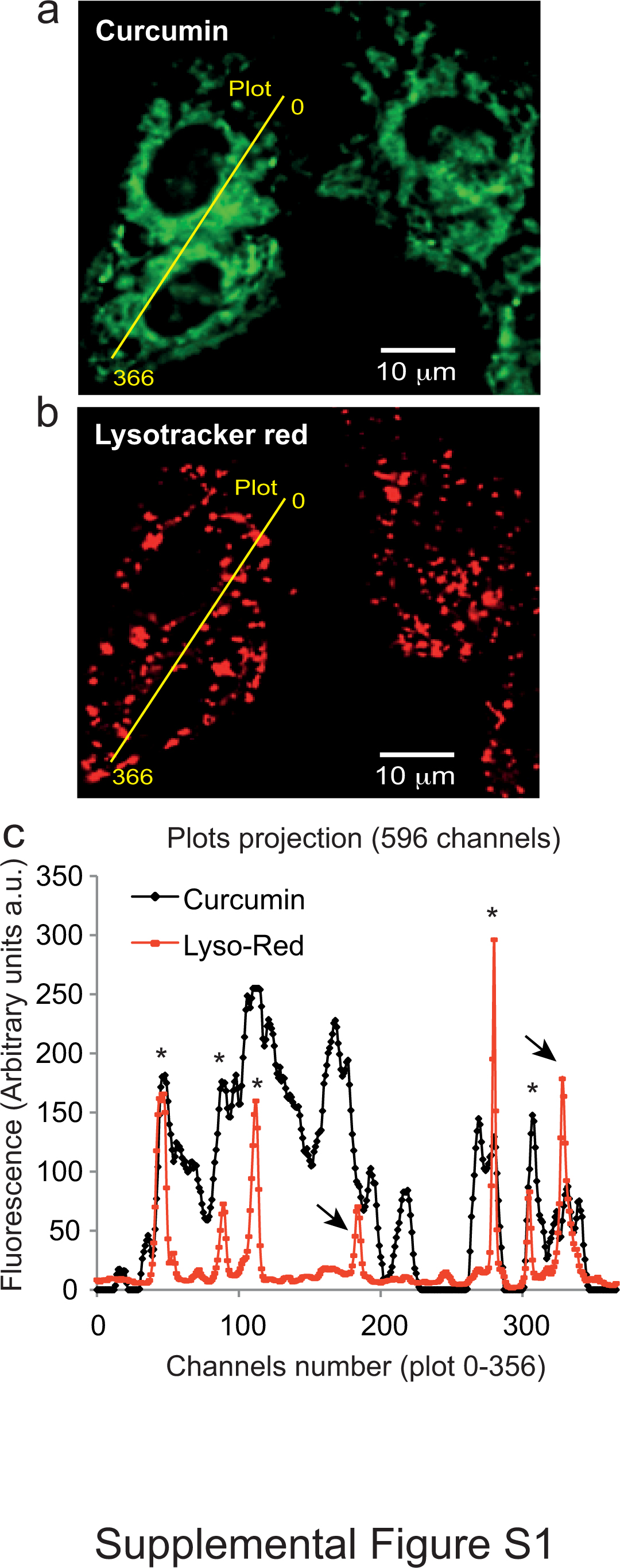

Supplement: Supplementary Figrue S1 [file cddiscovery201517-s1.jpg]

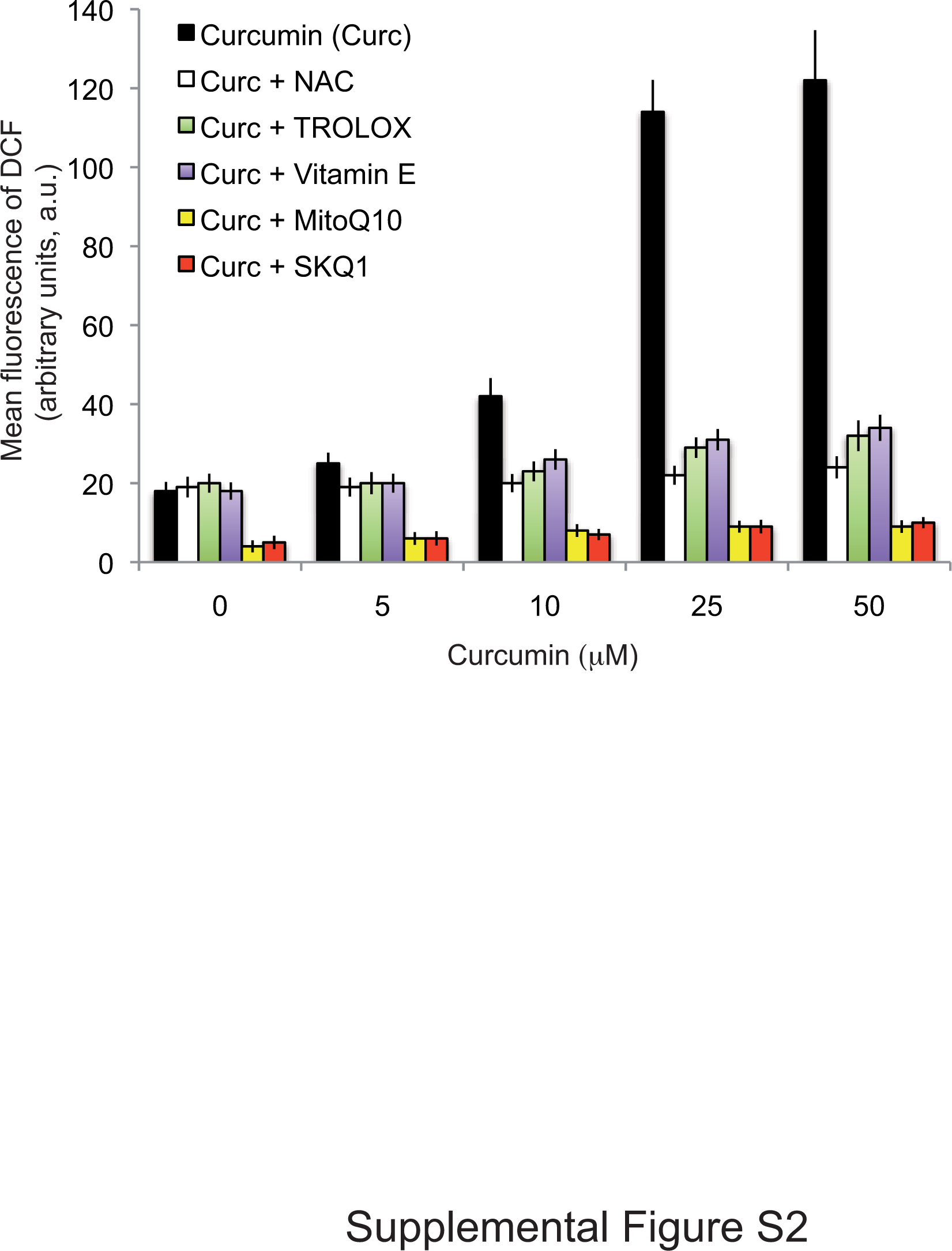

Supplement: Supplementary Figrue S2 [file cddiscovery201517-s2.jpg]

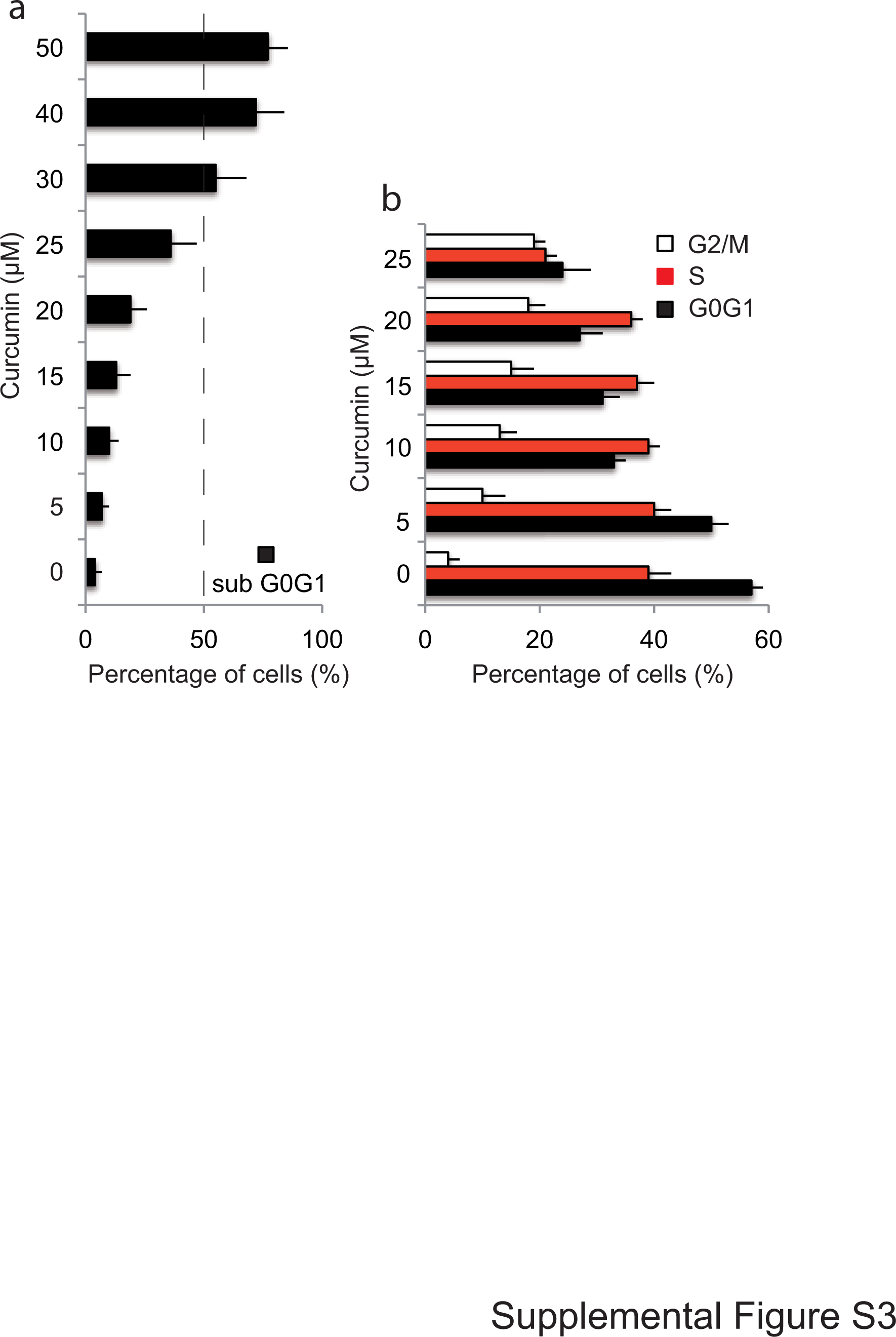

Supplement: Supplementary Figrue S3 [file cddiscovery201517-s3.jpg]

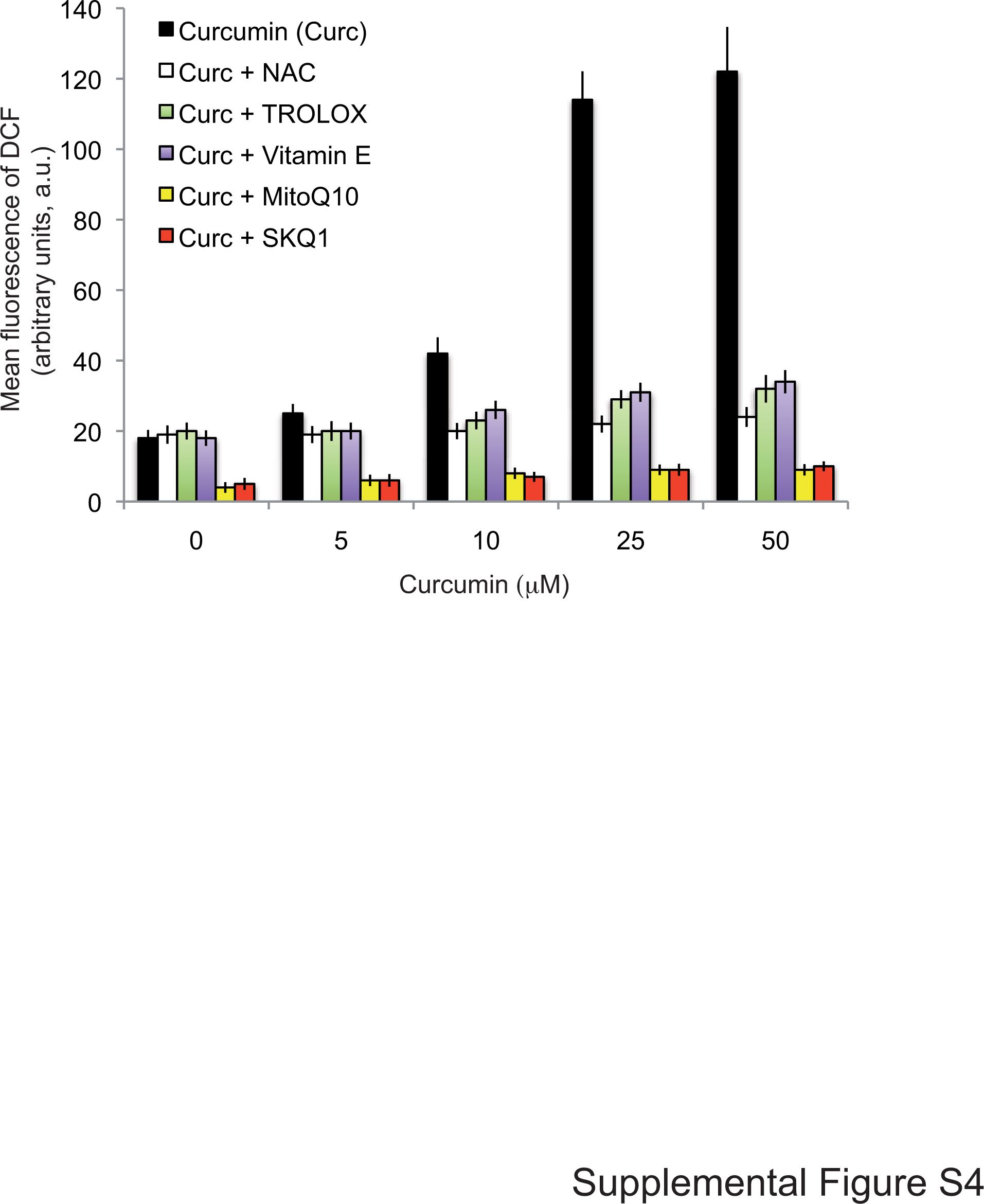

Supplement: Supplementary Figrue S4 [file cddiscovery201517-s4.jpg]
