# Supplementary material for: Curcumin induces crosstalk between autophagy and apoptosis mediated by calcium release from the endoplasmic reticulum, lysosomal destabilization and mitochondrial events
Source: Cell Death Discov. 2015 Oct 26;1:15017–. doi: 10.1038/cddiscovery.2015.17 (PMC4979459; doi:10.1038/cddiscovery.2015.17)
Supplement: Supplementary Figure Legends [file cddiscovery201517-s5.doc]

***Supplemental Figure S1. Localization of curcumin and lysotracker Red***

(a-c) - (a) Curcumin fluorescence at 530 ± 30 nm after excitation at 488 nm. (b) The lysosomal compartment was stained with lysotracker red and the picture was taken at 585 ± 20 nm after excitation at 488 nm. (c) Plot of fluorescence along the yellow line of 366 pixels shown in the picture. Curcumin is shown with a black line and lysotracker red staining with a red line. Black stars indicate strict co-localization and black arrows indicate no co-localization.

***Supplemental Figure S2. Effects of various ROS inhibitors on the fluorescence of the hydroperoxide marker, Dichlorofluorescein-diacetate.***

NAC, N-acetyl cysteine, TROLOX, Vitamin E and two mitochondrially targeted antixoidants, MitoQ10 and SKQ1, were used.
